# Body recomposition in adults: mechanisms, modulators, and practical algorithms for simultaneous skeletal muscle mass gain and fat mass loss

**DOI:** 10.1080/15502783.2026.2743880

**Published:** 2026-11-10

**Authors:** Siavash Naddafha, Brad J. Schoenfeld, Jose Antonio, Jeffrey R. Stout

**Affiliations:** a School of Medical and Health Sciences, Edith Cowan University, Joondalup, Australia; b Department of Exercise Science and Recreation, CUNY Lehman College, Bronx, NY, USA; c College of Osteopathic Medicine, Nova Southeastern University, Davie, FL, USA; d School of Kinesiology and Rehabilitation Sciences, University of Central Florida, Orlando, FL, USA

**Keywords:** Body fat, diet, body composition, skeletal muscle, muscle protein synthesis, muscle hypertrophy, dietary proteins, energy deficit, sarcopenic obesity, mTOR

## Abstract

**Background:**

Body recomposition is the concurrent increase in fat-free mass (FFM) or skeletal muscle mass and decrease in fat mass (FM) within the same intervention period. Although hypertrophy is commonly pursued in energy surplus and FM loss in energy deficit, controlled trials indicate that recomposition can occur when progressive resistance training (PRT), adequate protein intake, and an appropriate energy prescription are combined.

**Methods:**

We conducted a structured narrative review of randomised trials, meta-analyses, and selected mechanistic studies in adults, with emphasis on FFM, FM, strength, and performance outcomes. Device heterogeneity was considered when interpreting evidence, with greatest weight placed on four-compartment and dual-energy X-ray absorptiometry (DXA) outcomes.

**Results:**

PRT combined with higher protein intake, typically ~1.6-2.4 g/kg/day and higher relative intakes during energy restriction or leanness, is the most consistent strategy for recomposition. Effects are largest in untrained individuals and those with higher adiposity, smaller in trained or lean athletes, and still feasible in older adults when anabolic resistance is addressed with supervised PRT and sufficient protein. Moderate deficits, carbohydrate allocation around key sessions, sleep, and recovery support FFM retention or accretion while facilitating FM loss. Practical applications: Programmes should pair progressive overload with protein-centred diets, modest energy deficits, and periodic monitoring of body composition and performance.

**Conclusions:**

Body recomposition is feasible but context-dependent; expected changes are generally modest in trained or lean populations and require individualised adjustment.

## Key points

- Protein plus progressive resistance training are foundational. Daily protein intakes ≈1.6-2.4 g·kg^−1^, and higher relative intakes when lean or in energy deficit, with 0.3−0.5 g·kg^−1^ per meal, support recomposition.
- Keep deficits moderate. Aim for ~10-20% below maintenance, ≤0.5−0.7% body mass·wk^−1^ in trained individuals and slightly faster early in higher-adiposity novices, to bias weight change toward FM loss rather than FFM loss.
- Carbohydrate periodisation supports performance. Slightly higher carbohydrate intake on key training days can sustain intensity and support FFM retention; macronutrient ratio is secondary to protein adequacy, energy control, and adherence.
- Context dictates magnitude. Novices/high-BF clients typically see the largest recomposition; trained/lean athletes and older adults can achieve smaller but meaningful changes with meticulous programming.
- Monitoring. Combine body composition assessment, ideally DXA when feasible, with strength and performance metrics; adjust energy intake, protein targets, and training load when trends diverge from the desired ΔFFM and ΔFM pattern.

## 1. Introduction

Achieving simultaneous skeletal muscle mass gain and fat mass loss, often termed body recomposition, is traditionally viewed as difficult because classical models link hypertrophy to positive energy availability and FM loss to negative energy balance. Nevertheless, randomised controlled trials and controlled interventions show that concurrent increases in FFM and decreases in FM can occur with appropriate training and nutrition [1–6]. For clarity, this review defines body recomposition operationally as an increase in FFM (ΔFFM > 0) with a decrease in FM (ΔFM < 0) over the same intervention period [7]. Because most intervention studies quantify FFM rather than skeletal muscle mass directly, FFM and FM are used for measured body composition outcomes; skeletal muscle mass is used only when the cited evidence or interpretation specifically concerns muscle tissue.

Aims and Scope: Weight-stable recomposition denotes ΔFFM > 0 with ΔFM < 0 alongside negligible net body-mass change, interpreted within method-specific least-significant-change thresholds. This review synthesises nutrition, resistance training, concurrent training, and monitoring strategies relevant to recomposition in adults. We prioritise randomised controlled trials, meta-analyses, and selected mechanistic studies, and we translate the evidence into practical algorithms stratified by training status, adiposity, age, and sex. Relative to prior syntheses, including physique-athlete nutrition guidelines and systematic reviews [6,8,9], this review formalises the operational definition of recomposition and integrates nutrient-training decision rules for applied settings.

The evidence base has progressed from proof-of-principle trials under hypocaloric, higher-protein conditions, to studies in trained cohorts and older adults, and now to meta-analyses quantifying high-protein energy restriction and energy deficiency during resistance training (RT). Collectively, these data support a mechanism-informed but population-specific view of recomposition rather than a single universal protocol [1,4,9–13].

## 2. Methods

We performed a narrative review with systematic features and report in alignment with PRISMA 2020 and SWiM guidance for narrative syntheses. PubMed/MEDLINE, Embase, SPORTDiscus, Web of Science, and Cochrane CENTRAL were searched from inception to September 2025 for human studies on body composition change (fat-free mass [FFM], fat mass [FM]) in response to nutrition and/or exercise. The search combined terms for body composition, resistance/aerobic/concurrent training, protein, energy restriction, nutrient timing, and recomposition. We prioritised randomised controlled trials and meta-analyses; controlled trials and mechanistic tracer studies were included selectively to anchor protein dose/timing, energy-deficit responses, and concurrent-training considerations. Inclusion focused on adults (≥18 y), healthy or with overweight/obesity or athletic cohorts, interventions ≥2 weeks, and body composition outcomes measured by dual-energy X-ray absorptiometry (DXA), air displacement plethysmography (ADP), magnetic resonance imaging (MRI), computed tomography (CT), multi-frequency bioelectrical impedance analysis (BIA), bioimpedance spectroscopy (BIS), skinfolds, or ultrasound. Exclusions were paediatric populations, pregnancy, pharmacologic anabolic use, bariatric surgery, severe catabolic disease, and non-original reports. Primary outcomes were ΔFFM and ΔFM; secondary outcomes included strength and performance [12,14,15]. Given heterogeneity, we conducted a structured narrative synthesis rather than a quantitative meta-analysis.

### 2.1. Quality appraisal

#### 2.1.1. Quality appraisal and device interpretation

For RCTs, we evaluated randomisation sequence generation, allocation concealment, baseline balance, assessor blinding for body composition outcomes, completeness of outcome data, and intention-to-treat analyses. For non-randomised trials, assessments focused on confounding, selection bias, diet verification, training supervision, and intervention fidelity. Each study was graded as low risk, some concerns, or high risk of bias, and conclusions were weighted toward studies with stronger design, better adherence verification, and standardised body composition protocols.

To address device heterogeneity, we applied a pragmatic interpretation framework rather than a rigid evidentiary hierarchy. Four-compartment models were considered criterion-level estimates for whole-body FFM and FM. DXA was weighted highly because of its availability and reproducibility under standardised conditions, whereas ADP, BIA, BIS, skinfolds, and ultrasound were interpreted according to the population, device model, hydration status, fasting status, recent exercise, and protocol control. This ordering does not imply that ADP or BIA is universally superior to BIS; comparative validity is context-dependent and should be judged against appropriate criterion measures. Distinct from whole-body compartment estimates, MRI and CT were considered criterion approaches for regional skeletal muscle cross-sectional area or volume. We did not pool outcomes across device classes, and inadequate standardisation led to lower confidence in the study-level inference [16,17].

A PRISMA-style flow diagram summarising identification, screening, eligibility, and inclusion is presented in Figure 1.

**Figure 1.**
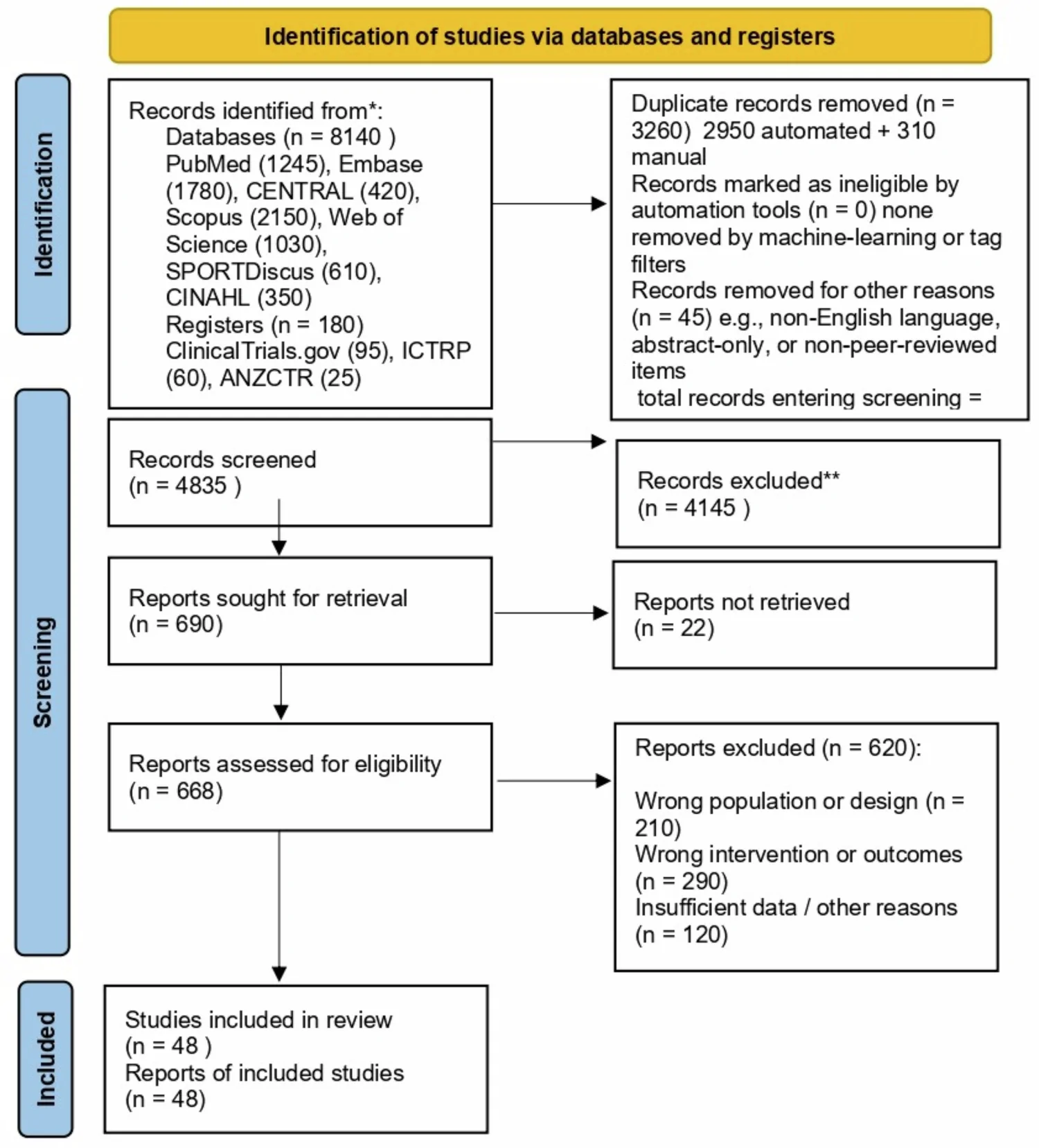
PRISMA-style flow diagram summarising database searches, duplicate removal, screening, eligibility assessment, and included studies.

### 2.2. Mechanisms of skeletal muscle hypertrophy in energy surplus and deficit

Muscle hypertrophy results from a chronic net positive muscle protein balance, meaning muscle protein synthesis (MPS) exceeds muscle protein breakdown (MPB) over time. Resistance exercise is the primary stimulus for elevating MPS, especially when combined with protein feeding [5]. Mechanistically, RT initiates intracellular signalling cascades (e.g. mTOR pathway activation) that upregulate MPS, leading to the addition of contractile proteins and hence muscle fibre growth. Sufficient dietary protein provides essential amino acids to maximise this MPS response. In young adults, ingesting ~20–40 g of high-quality protein per meal stimulates robust MPS, and distributing protein feedings evenly (e.g. 3–5 meals/day) may optimise the anabolic response over 24 hours [18]. Over days and weeks of training, repeated bouts of MPS that exceed MPB translate into accrued FFM and skeletal muscle mass. Dose response work further suggests a per-meal target of ~0.24–0.40 g·kg^−1^ in young adults (∼2–3 g leucine), whereas older adults exhibit anabolic resistance and may require ~0.40–0.60 g·kg^−1^ per meal of high-leucine protein to maximise MPS; “fast” whey produces a larger acute MPS response than casein, while “slow” casein supports prolonged aminoacidemia [5,19].

Traditionally, a caloric surplus is viewed as conducive to FFM gains because energy availability can support the energetically costly process of tissue accretion. A surplus also tends to raise circulating insulin and IGF-1, hormones that can promote anabolism. However, muscle hypertrophy per se is not a process that requires large amounts of extra energy on a day-to-day basis. The energetic cost of synthesising new skeletal muscle is relatively modest; for example, accruing 1 kg of skeletal muscle is estimated to require approximately 1,800-2,500 kcal above maintenance distributed over the growth period, which is lower than the energy cost of adipose-tissue deposition [20]. Thus, a large energy surplus is not necessary for FFM and can lead to disproportionate fat gain [21]. Muscle fibres can draw on internal energy (intramuscular triglycerides, glycogen) and systemic fuels to support hypertrophy if training and protein intake are sufficient [22]. Evidence from isotope tracer studies indicates that during caloric restriction, resistance exercise still stimulates MPS, though to a somewhat blunted magnitude if protein intake is inadequate [5]. With high protein availability, the body can utilise dietary amino acids for muscle repair while mobilising stored fat to meet energy needs, permitting hypertrophy even in an overall catabolic environment [4]. During caloric restriction, the responsiveness of MPS and the endocrine milieu are blunted reflecting “anabolic resistance” which attenuates FFM accretion even when RT is performed; meta-regression suggests energy deficits of approximately ~ 500 kcal·d^−1^ may negate FFM gains in trained individuals [12].

Muscle protein breakdown (MPB) plays a paradoxically important role in body recomposition. Although often framed as counterproductive to hypertrophy, MPB enables the selective removal of damaged proteins and contributes to remodelling when muscle protein synthesis (MPS) exceeds breakdown over time. Methodological constraints in human research also limit the resolution of MPB data relative to MPS [23]. Available evidence suggests that resistance exercise increases MPB, but this increase is smaller than the concurrent rise in MPS, identifying synthesis as the primary driver of net anabolic balance. Consequently, nutritional strategies for recomposition should prioritise maximising MPS through adequate protein intake and resistance training rather than attempting to suppress MPB indiscriminately [11].

In summary, skeletal muscle hypertrophy fundamentally depends on the MPS > MPB relationship and can occur in energy balance or even in a deficit. In drug-free, eugonadal populations, it is most reliably achieved with the performance of progressive RT in combination with sufficient protein intake; however, exogenous anabolic-androgenic steroids can induce appreciable increases in fat-free mass, even without RT or deliberately high protein, and may preserve FFM during a severe energy deficit. Accordingly, our conclusions pertain to non-pharmacological (drug-tested/health) contexts [24]. The mechanisms enabling hypertrophy during a deficit include prioritisation of dietary amino acids to muscle repair, improved nutrient partitioning (the body preferentially drives nutrients to muscle if the stimulus is present), and drawing upon fat stores to fuel the energy cost of MPS [4,22]. Higher dietary protein intake is a key “nutrient driver” in this context as it provides substrate for MPS and has a muscle-sparing effect. In a hallmark RCT by Longland et al. [4], young adults engaged in intense training while consuming either ~2.4 g/kg or ~1.2 g/kg protein in a 40% calorie deficit. After 4 weeks, the high-protein group gained 1.2 kg FFM, compared to ~0.1 kg in the normal protein group, despite both losing significant weight [4]. This demonstrates that robust hypertrophy can occur in an energy deficit when protein is sufficiently high and a training stimulus is present. The physiological machinery for muscle growth remains operative in a deficit, albeit requiring greater attention to protein and recovery.

### 2.3. Mechanisms of fat loss and influence of nutrition

Reduction of adipose tissue requires a sustained period where energy expenditure exceeds intake, forcing the body to mobilise stored triglycerides to meet needs for sustenance. Lipolysis (the breakdown of triglycerides into free fatty acids and glycerol) is stimulated by an energy deficit and hormonal signals (e.g. increased catecholamines, lowered insulin). Free fatty acids are then oxidised in tissues (especially muscle and liver) to yield ATP, resulting in a loss of fat mass over time. A core mechanism of fat loss is therefore an energy imbalance: creating a negative energy balance via diet, exercise, or both will trigger fat catabolism [2]. The magnitude of fat loss is roughly proportional to the cumulative energy deficit (within practical limits), with ~7700 kcal deficit needed per kilogram of fat lost, though individual efficiency varies [25].

Diet composition can modulate the efficiency of fat loss and the preservation of FFM during weight loss [26]. Higher-protein diets have a well-documented advantage in weight loss interventions: they increase satiety, elevate thermogenesis (diet-induced thermic effect), and crucially, reduce the loss of fat-free mass compared to carbohydrate- or fat-heavy diets [9]. Protein has a higher thermic effect (~20-30% of its calories used in digestion) versus fats or carbohydrate (~5-15%), meaning a high-protein diet yields a slightly greater energy deficit for the same caloric intake. Moreover, protein and amino acids (especially leucine) directly stimulate MPS, helping maintain muscle even as fat is lost [5]. For example, Mettler et al. [11] showed that doubling protein intake (to ~2.3 g/kg) in athletes halved the loss of FFM over 2 weeks of energy restriction, without affecting the magnitude of fat loss compared to ~1.0 g/kg protein. Although this short-term intervention did not result in net FFM accretion, it demonstrated that higher protein intake significantly attenuated FFM loss compared to the control condition. Thus, while a caloric deficit underpins fat loss, the nutrient profile, particularly ample protein, ensures that weight loss is partitioned primarily towards adipose tissue reduction rather than FFM degradation [4,22].

### 2.4. Exercise energy expenditure further facilitates fat loss

Aerobic exercise (endurance training) increases total caloric output and can preferentially draw upon fat stores, especially when performed at moderate intensities or for longer durations [27,28]. Regular endurance training improves mitochondrial capacity and fat oxidation, which over time contributes to reductions in fat mass [28]. RT, though primarily an anabolic stimulus, also contributes to energy expenditure and can induce fat loss. Notably, resistance exercise can create an “afterburn” effect (excess post-exercise oxygen consumption) However, EPOC contributes a relatively small fraction of daily energy expenditure; training quality and total weekly workload remain primary drivers of outcomes [29]. and promotes favourable hormonal changes (e.g. transient growth hormone elevations) that may assist fat metabolism. A systematic review by Willis et al. [28] found that aerobic exercise tends to produce larger absolute fat mass reductions than RT in weight-loss programmes for untrained individuals, but the combination of resistance and aerobic training is most effective for improving body composition In practice, combining modalities often yields the best recomposition: RT provides the mechanical tension to stimulate FFM accretion, moderate-intensity aerobic training maximises total energy expenditure and acute lipid oxidation, while high-intensity interval training (HIIT) offers time-efficient caloric output and improvements in oxidative capacity, with diet controlling net energy balance.

### 2.5. Role of carbohydrate and fat intake

For fat loss, the relative proportion of dietary carbohydrate vs. fats is less important than the caloric deficit and protein sufficiency, as numerous trials have shown successful fat loss on both low-carb and low-fat regimens [30]. However, extreme approaches can have specific effects: very low-carbohydrate ketogenic diets may accelerate short-term water loss and suppress appetite, aiding fat loss, but they can also potentially compromise high-intensity training performance due to limited glycogen, which might indirectly affect muscle retention or gain. Conversely, low-fat high-carb diets can support intense training but may be more difficult for appetite control in some individuals. For recomposition, a balanced approach may work best: moderate carbohydrate to fuel training performance (and thus better muscle stimulus) and sufficient dietary fat to maintain hormonal function, all within an overall hypocaloric framework that prioritises protein. Preservation of training intensity during energy restriction is critical for maintaining fat-free mass. Carbohydrate periodisation, i.e. providing greater carbohydrate availability on high-load training days and reducing intake on rest or low-load days, may support glycogen replenishment and performance while maintaining a net weekly energy deficit [15,28].

Metabolic adaptations during prolonged energy restriction, compensatory reductions in resting metabolic rate and increases in hunger collectively termed adaptive thermogenesis can attenuate ongoing fat loss [26,31]. RT helps blunt these effects primarily by preserving fat-free mass, thereby maintaining resting energy expenditure and training capacity [28,32]. High-protein diets can further mitigate adaptive thermogenesis because protein has the highest diet-induced thermogenesis and supports retention of FFM, both of which help sustain energy expenditure during dieting [33]. Fuel partitioning is also pivotal: in a deficit, whether energy is derived predominantly from adipose tissue versus amino acids depends on deficit size, macronutrient composition, and the presence of an exercise stimulus. Smaller daily deficits (~300–500 kcal) combined with RT typically bias losses toward fat rather than FFM [12,28], whereas very large deficits increase the risk of proteolysis; moreover, the leaner the individual, the greater the proportion of FFM typically lost for a given weight change [31,32]. Collectively, sustained fat loss depends on an energy deficit, but dietary protein and RT materially influence how that weight is partitioned enabling preferential fat loss and, in some contexts, concurrent FFM gain.

### 2.6. Modulators of body recomposition: training status, initial body composition, and age

Capacity for recomposition varies by training status, starting adiposity, age, sex, sleep, and hormonal milieu. These factors shape both the magnitude of ΔFFM and ΔFM and the aggressiveness of the nutrition and training prescription.

## 3. Training status: untrained/novice vs. trained individuals

### 3.1. Untrained and novice individuals

For this review, untrained or novice individuals are defined as adults with no structured progressive resistance training for ≥ 12 months or no prior exposure to systematic hypertrophy-oriented programming. Simultaneous FM loss with FFM accretion is most prevalent in this group because early RT elicits large increases in muscle protein synthetic sensitivity, rapid neural adaptations, and favourable energy partitioning, especially when paired with higher protein intakes during weight loss. Randomised trials in overweight, previously sedentary adults show that coupling hypocaloric diets with RT and higher protein, often via dairy, produces greater FM loss and measurable FFM gains compared with lower-protein approaches [3]. Contemporary narrative reviews similarly conclude that recomposition is common in untrained and higher-adiposity populations under structured RT with adequate protein [7].

### 3.2. Trained individuals and athletes

For this review, trained individuals are defined as adults with ≥1 year of consistent progressive resistance training and established proficiency with multi-joint resistance exercises; athletes are considered trained when sport preparation includes regular resistance training or body composition management. In resistance-trained populations, recomposition is less common and generally smaller in magnitude, but it remains achievable with meticulous programming and nutrition. In resistance-trained women following an 8-week lifting programme, a high-protein diet (~2.5 g·kg^−1^·d^−1^) produced larger increases in FFM and greater FM loss than a moderate-protein diet [1]. More broadly, a meta-analysis indicates that sustained energy deficiency blunts FFM gains from RT while often preserving strength, supporting modest deficits or near-maintenance intakes when recomposition is the goal in advanced training states [12]. Case studies of physique-sport preparation indicate that high protein and resistance training can preserve, and occasionally increase, regional FFM during early weight loss, but these effects are often compromised at very low adiposity [34]. Collectively, novices have a larger anabolic reserve, whereas trained individuals require tighter control of energy balance, high-quality RT, adequate protein, sleep, and careful fatigue management (Tables 1 and 2).

**Table 1.** Recomposition playbook by population, split across Panels A-C for readability.

A. Expected response, rate of weight change, energy target, and protein target				
Population	Expected magnitude (12–16 wk)	Weekly weight-change target	Energy target	Protein targets
Novice (untrained)	FFM + 1–3 kg; FM −2–6 kg; strength increase markedly	−0.5–7.0% BM·wk^−1^ (≤1.0% if high BF)	−10–20% (up to −25% if high BF, taper as you lean)	Daily: 1.8–2.4 g·kg^−1^·d^−1^; per-meal: 0.30–0.40 g·kg^−1^ across 6–5 feedings
Trained (experienced)	FFM 0–1.5 kg; FM −1–3 kg (small but meaningful)	−0.25–0.5% BM·wk^−1^	−5–15% or zig-zag around maintenance (±5–10%)	2.0–2.7 g·kg^−1^·d^−1^ (up to ~3.1 g·kg^−1^ FFM when very lean); per-meal 0.30–0.50 g·kg^−1^ across 4–6 feedings
High body fat (men ≥ 20%; women ≥ 30%)	FFM 0–3 kg; FM −4–10 kg	−0.7–1.0% BM·wk^−1^	−15–25% (reduce as leanness improves)	1.8–2.4 g·kg^−1^·d^−1^; per-meal 0.30–0.40 g·kg^−1^ across 5–6 feedings
Low body fat/lean (men ≤ 12%; women ≤ 22%)	FFM −0.5 to + 0.5 kg; FM −0.5–2 kg; performance = KPI	−0.25–0.5% BM·wk^−1^ (or weight-stable)	−5–10% or near-maintenance	2.2–2.7 g·kg^−1^·d^−1^; (≈2.5–3.1 g·kg−1 FFM at 12% body fat) per-meal 0.40–0.50 g·kg^−1^ across 5–6 feedings (leucine-rich)
Adults (18–59)	FFM 0–2 kg; FM −1–4 kg	−0.5–0.7% BM·wk^−1^	−10–20%	1.6–2.0 g·kg^−1^·d^−1^ (higher if deficit > 15%)
Older adults (≥60)	Preserve or FFM + 0–1 kg; FM −1–3 kg; function increase	−0.25–0.5% BM·wk^−1^ (avoid aggressive cuts)	−10–15% (often start at maintenance)	1.2–1.8 g·kg^−1^·d^−1^; across 3–4 feedings; per-meal 0.40–0.60 g·kg^−1^; leucine-rich; pre-sleep casein 30–40 g optional
Women	FFM 0–1.5 kg; FM −1–4 kg; performance often maintained	−0.3–0.7% BM·wk^−1^	−10–20% (conservative if cycle disruption)	1.8–2.4 g·kg^−1^·d^−1^; iron/calcium adequacy
Men	FFM 0–2 kg; FM −1–4 kg	−0.5–0.7% BM·wk^−1^	−10–20%	1.8–2.4 g·kg^−1^·d^−1^

B. Carbohydrate, fat, aerobic training, and resistance-training priorities				
Population	Carbohydrate strategy	Fat minimum	Cardio dose & modality (sequencing)	Resistance-training priorities
Novice (untrained)	Train days 3–5 g·kg^−1^; rest 2–3 g·kg^−1^; front-load pre/post RT	≥0.6–0.8 g·kg^−1^ or ≥ 20–30% kcal	2–3 × ·wk^−1^ total: HIIT ≤ 2 × (10–15 min), MICT 20–30 min; 8–12k steps·d^−1^; do RT - cardio or separate by ≥ 3 h	Learn technique; full-body 3 × ·wk^−1^; 10–15 sets·muscle^−1^·wk^−1^; proximity to failure (RPE 7–9)
Trained (experienced)	Heavy days 3–6 g·kg^−1^; keep carbohydrate high on key sessions; 24–48 h carb-dominant refeeds before testing/heavy volume	≥0.6–0.8 g·kg^−1^	2–3 × ·wk^−1^ low-impact (cycle/row); minimise long runs if leg hypertrophy; separate from RT or do after	Anchor heavy work ≥ 75–90% 1RM; 10–20 sets·muscle^−1^·wk^−1^; track performance; manage fatigue with deloads
High body fat (men ≥ 20%; women ≥ 30%)	2–4 g·kg^−1^; centre carbohydrate around RT; fibre-rich, lower-GI staples	≥0.6–0.8 g·kg^−1^	3–4 × ·wk^−1^ mixed: HIIT 1–2×; MICT 20–40 min; 10–12k steps·d^−1^	RT volume moderate-high; multi-joint emphasis; progressive overload
Low body fat/lean (men ≤ 12%; women ≤ 22%)	Keep 3–6 g·kg^−1^ on lift days; 1–2 refeed days·wk^−1^ tied to hardest sessions	≥0.8–1.0 g·kg^−1^ if very lean	1–2 × ·wk^−1^ low-impact; brief HIIT finishers only	Retain heavy exposures; reduce junk volume; guard bar speed and performance
Adults (18–40)	Train-day emphasis; peri-workout carbohydrate	≥0.6–0.8 g·kg^−1^	2–3 × ·wk^−1^ any modality matched to recovery	Progressive overload non-negotiable; periodise
Older adults (≥60)	Even distribution; moderate-GI carbohydrate to support RT	≥0.8 g·kg^−1^	2–3 × ·wk^−1^ MICT 20–40 min + daily steps; add balance/power drills	RT 3 × ·wk^−1^; multi-joint + power (light-load, fast intent); supervised when possible
Women	3–5 g·kg^−1^ with peri-workout focus; higher carbohydrate in high-intensity microcycles	≥0.6–0.8 g·kg^−1^	2–3 × ·wk^−1^ per preference; low-impact if leg growth prioritised	Similar hypertrophy dosing; accommodate cycle-related fatigue
Men	Heavy/lower-body days 3–6 g·kg^−1^; pre/post RT timing	≥0.6–0.8 g·kg^−1^	2–3 × ·wk^−1^; minimise interference	Heavy anchors + targeted volume

C. Refeeds, monitoring indicators, and red flags			
Population	Refeeds/diet breaks	Monitoring KPIs	Red flags/notes
Novice (untrained)	Optional 1–2 d/wk maintenance-calorie refeeds to support adherence/performance	DXA/ADP if available; waist decreased; 5RM/volume increased; sleep 7–9 h	Avoid crash cuts; rapid HIIT ramp; consider creatine 3–5 g·d^−1^
Trained (experienced)	Strategic 24–48 h refeeds; diet breaks (5–7 d @ maintenance) each 4–6 wk if performance fades	Strength preservation or increased on key lifts; HRV/s-RPE stable; sleep/mood stable	Overreaching signs greater shrink deficit first (Murphy & Koehler)
High body fat (men ≥ 20%; women ≥ 30%)	Refeeds ad hoc for adherence; avoid “cheat days”	Waist decreased; DXA/ADP where feasible; step counts maintained	Micronutrient sufficiency; avoid sudden HIIT spikes
Low body fat/lean (men ≤ 12%; women ≤ 22%)	Planned refeeds; diet breaks every 3–4 wk if performance/HRV decline	Performance stable/decreased; bar velocity; visual leanness	Menstrual disturbance, sleep issues, libido decreasing shrink deficit; consider maintenance phase
Younger adults (18–40)	Refeed around peaks/tests	5RM/volume decreased; body comp trend	Alcohol/sleep debt blunt gains
Older adults (≥60)	Use maintenance weeks more frequently	Chair-rise speed, grip, gait speed; appendicular FFM	Sarcopenia risk - vitamin D sufficiency; consider creatine
Women	Place refeeds in high-demand weeks	Cycle regularity, HRV, performance	REDs risk; monitor energy availability; treat LEA early
Men	Short refeeds for performance	Strength KPIs; waist decreased	Excess HIIT + low carbohydrate greater strength loss; limit alcohol; sleep ≥ 7 h

**Abbreviations**: BM, body mass; FFM, fat-free mass; FM, fat mass; RT, resistance training; HIIT, high-intensity interval training; MICT, moderate-intensity continuous training; HRV, heart-rate variability; REDs/LEA, Relative Energy Deficiency in Sport/Low Energy Availability; DXA, dual-energy X-ray absorptiometry; ADP, air-displacement plethysmography.

Note: Values reflect typical responses under structured RT with adequate sleep and adherence. Protein bands and per-meal targets align with consensus for athletes and during hypocaloric phases. Older adults require higher per-meal doses to overcome anabolic resistance. Rate of loss: in trained or lean individuals, keep ≤0.5% body mass·wk^−1^; in high-BF novices, ≤1% is generally tolerated. Concurrent aerobic work does not inherently blunt hypertrophy when modality, sequencing, and volume are managed. Creatine (3–5 g·d^−1^) supports training quality and **FFM retention**. Abbreviations: FFM fat-free mass; FM fat mass; RT resistance training; HIIT high-intensity interval training; MICT moderate-intensity continuous training; LBM lean body mass; REDs Relative Energy Deficiency in Sport; GI glycemic index.

**Table 2.** Recomposition-relevant intervention evidence, split across Panels A-B for readability.

A. Intervention characteristics and body composition assessment method								
Context/exemplar	n	Sample & training status	Energy prescription	Protein (g·kg^−1^·d^−1^)	RT prescription	Aerobics/conditioning	Duration	Method
Large deficit + intense training [4]	40	Young, recreationally active, untrained	~40% deficit	High ≈ 2.4 vs Moderate ≈ 1.2	Full-body RT + intervals	HIIT integrated	4 wk	4-Compartment (4C) Model
Physique-trained women [1]	27	Resistance-trained women	Ad lib/mild deficit	≈ 2.5 vs ≈ 0.9	Periodised hypertrophy	Light cardio allowed	8 wk	Skinfolds & B-mode Ultrasound
Overweight women (novice) [3]	90	Overweight, untrained	Hypocaloric	Higher dairy/protein vs adequate	Circuit-style + EX	MICT added	16 wk	DXA
Aggressive short cut [11]	20	Trained men athletes	~40% deficit	≈ 2.3 vs ≈ 1.0	Habitual RT	Minimal	2 wk	DXA
Older adults [13]	100	Overweight/obese, 55–80 y	Hypocaloric	1.3 vs ~0.8; with/without RT	3 × ·wk^−1^ supervised	Walking added	10 wk	Air Displacement (BodPod)
Supplemented older adults [35]	80	Obese, community-dwelling	Hypocaloric	Whey + leucine + Vit-D vs iso-cal	RT + functionals	Walking	~13 wk	DXA (ALA)
Elite athletes, rate of loss [14]	24	Elite, mixed sex	Slow ~0.7%·wk^−1^ vs Fast ~1.4%·wk^−1^	Adequate	Structured RT	Sport-specific	8–12 wk	DXA
Macro split over 12 months [30]	609	Overweight adults	Behaviour-guided deficit	LF vs LC (adequate protein)	Habitual	Habitual	12 mo	DXA/subsets
High protein dieting meta-analysis [9]	24 trials	Mixed BMI	Hypocaloric	Higher vs standard	Mixed	Mixed	,	Mixed
Energy deficiency in trained [12]	26 studies	Trained	Deficit vs neutral	Varied	Standardised RT	,	,	Mixed
Across lifespan with overweight/obesity [8]	116 studies	Youth>older; overweight/obesity	Mixed	Mixed	RT emphasis	Mixed	—	Mixed
Intermittent vs continuous dieting in resistance-trained ([36], MSSE)	61	Resistance-trained adults	IER (1-wk diet breaks) vs continuous, matched deficit	~2 g·kg^−1^	Periodised RT	Standardised	12–15 wk	DXA
Diet-break secondary outcomes ([37], PLoS ONE)	26	Same cohort as above ( Intermittent-arm completers from the same trial)	same	~2 g·kg^−1^	Periodised RT	—	12-wk block	DXA (secondary)

B. Body-composition and performance outcomes					
Context/exemplar	ΔFFM	ΔFM	Performance	Keynote	Design
Large deficit + intense training [4]	High: increased~1.2 kg	decreased (High > Mod)	Strength increased both	Proof-of-principle: FFM gain in deficit with high protein + hard RT	RCT
Physique-trained women [1]	High: increased~2.1 vs 0.6 kg	High PRO: decreased~1.1± 0.3 kg vs Low PRO: decreased-0.8 ±0.2 kg	1RM Squat & Deadlift Increased both (no group difference, *p* > 0.05)	Recomp in trained cohort superior FFM accretion with 2.5 g/kg/d protein	RCT
Overweight women (novice) [3]	HPHD: increased +0.4 to +0.7 kg vs APMD: 0.0 kg vs APLD: decreased -0.4 kg	HPHD: decreased −4.5 kg vs APMD: −3.8 kg vs APLD: −3.1 kg (p < 0.05)	Maximal strength (row, curl, leg ext) increased greatest in HPHD	HP group lost only fat while gaining FFM	3-armRCT
Aggressive short cut [11]	High PRO: decreased −0.3 kg LBM vs Control: decreased −1.6 kg LBM (p = 0.006)	Fat loss identical (~ −1.0 kg) between groups	Performance (1RM bench, Wingate, vertical jump)maintained	High protein (2.3 g/kg) spares >80% LBM loss during severe 2- wk energy deficit	Parallel RCT
Older adults [13]	+ 0.6 kg (HP + RT only)	−3–4 kg (all diet arms)	Strength increased (max in HP + RT)	Multimodal approach needed for FFM gain	4-arm RCT
Supplemented older adults [35]	Whey+Leucine+Vit-D: increased+0.37 kg AMM vs Control: decreased −0.53 kg (p=0.03)	~−3 kg both	Function increased	Leucine/Vit-D preserves appendicular lean	RCT
Elite athletes, rate of loss [14]	Slow Reduction (SR): increased +1.0 ± 0.2 kg vs Fast (FR): decreased −0.3 ± 0.4 kg	SR: decreased −4.9 ± 0.7 kg vs FR: decreased −3.2 ± 0.5 kg (greater absolute in Slow)	Strength/power better slow	Recommend slower cuts in trained	RCT
Macro split over 12 months [30]	12-mo change: −5.3 kg (Low- Fat) vs −6.0 kg (Low-Carb) (p = 0.20)	Similar	Fitness ↑ modestly	Macro ratio < deficit + protein for fat loss	2arm RCT (DIETFITS)
High protein dieting meta-analysis [9]	Weighted Mean Diff (WMD): +0.51 kg FFM retention (95% CI 0.03–1.00)	WMD: -0.87 kg greater Fat Mass loss (95% CI: −1.29 to −0.45)	Not quantitatively meta- analyzed	HP diets enhance fat loss & spare lean	Meta-analysis
Energy deficiency in trained [12]	500 kcal/d deficit completely ablates RT-induced LBM accretion	Fat mass loss proportional to energy deficit severity	Strength gains (1RM) fully preserved despite energy deficit	Energy deficits impair muscle hypertrophy but do not inhibit neural strength adaptations	Meta-analysis (26 studeis)
Across lifespan with overweight/obesity [8]	FFM increased (small–moderate)	FM decreased (moderate)	Strength & functional capacity improved	RT improves body comp across ages; effects persist with diet-induced loss	(114 trials) meta-analysis
Intermittent vs continuous dieting in resistance-trained ([36], MSSE)	Fat-free mass preserved similarly in mIER and mCER groups	Fat mass reduction similar between mIER (−3.2 kg) and mCER (−2.6 kg)	Appetite/satiety advantages; no REE benefit	Diet breaks improve hunger management and adherence without compromising recomposition	Parallel RCT (ICECAP)
Diet-break secondary outcomes ([37], PLoS ONE)	Fat-free mass transiently increased (+0.6 kg) during diet break	Fat mass unchanged during 1- week diet break	Leg muscle endurance (knee extension) increased significantly post-break	1-week diet break restores glycogen, improves muscle endurance, and reduces fatigue	Pre-specified analysis

Abbreviations: FFM, fat-free mass; FM, fat mass; RT, resistance training; MICT, moderate-intensity continuous training; HP, high protein; Mod, moderate protein; LF/LC, low-fat/low-carb; IER, intermittent energy restriction; REE, resting energy expenditure.

### 3.3. Initial body composition (body fat levels)

A person’s starting body fat percentage strongly influences how weight loss or gain is partitioned between fat mass and fat-free mass, often described by the Forbes theory of body composition change. Individuals with higher initial body fat tend to lose a greater proportion of weight from fat and less from FFM when dieting, and conversely, when gaining weight, they tend to put on more fat relative to muscle [31]. In contrast, very lean individuals (already low body fat) are at risk of losing more FFM in a deficit and, when overfeeding, their weight gain skews more towards increases in muscle [31]. This has direct implications for recomposition: those with higher body fat (e.g. overweight/obese individuals) have an easier time mobilising fat for fuel and can even support muscle anabolism with those fat-derived calories. Their bodies essentially have an energy surplus internally (stored fat) even when in an external caloric deficit. Indeed, many classic recomposition studies involve overweight subjects who, with exercise and high protein consumption, dramatically improve body composition. For example, in a trial of obese police officers, a combination of circuit training and a high-protein diet led to significant fat loss (~5–6% of body weight) while also increasing FFM (~2% gain) over 12 weeks [38]. High body fat appears to provide a “buffer” that spares protein tissue during weight loss meaning the fatter someone is, the more of the deficit can be met by fat stores [31]. Thus, overweight novice trainees often see the most striking recomposition, as they have both the reactivity of being untrained and the metabolic cushion of ample fat stores.

By contrast, lean individuals attempting recomposition operate near the limits of leanness, where the body is more protective of fat stores and the risk of FFM loss is higher during energy restriction. For instance, an athlete at 10% body fat attempting to reduce FM while increasing skeletal muscle mass faces a substantially narrower physiological margin than a higher-adiposity novice. This is why bodybuilders in contest preparation generally focus on preserving FFM as adiposity approaches very low levels [6]. Practically, the higher the starting adiposity, the more feasible recomposition tends to be; already-lean athletes usually require slower rates of loss, smaller deficits, and closer monitoring.

### 3.4. Age (young vs. older adults)

Aging affects body composition and the capacity for recomposition. Older adults (typically defined as > ~50–60 years) experience anabolic resistance of muscle a blunted muscle protein synthesis response to both resistance exercise and protein intake [19]. They also often have a propensity to gain fat and lose muscle with sedentary aging. These factors make simultaneous FFM accretion and FM loss particularly challenging in older populations, but possible. The presence of sarcopenic obesity (low skeletal muscle mass, high fat mass) in many older adults is a scenario where recomposition would be highly beneficial: reducing fat mass to improve metabolic health while increasing skeletal muscle mass to enhance functionality. Research indicates that older adults can indeed undergo recomposition with combined interventions. For instance, a 2017 randomised trial by Verreijen et al. [13] in overweight older adults (55–80 years) put participants on a 10-week hypocaloric diet. All lost weight, but only the group that received a higher protein diet (1.3 g/kg) plus thrice-weekly resistance exercise showed a statistically significant increase in fat-free mass (+0.6 kg), despite also losing ~3–4 kg of fat [13]. The other groups (diet alone or diet + either protein or exercise alone) lost fat but did not gain FFM. This underscores that in older adults, multimodal intervention is key: dietary protein, progressive (RT), and adequate total energy (not an overly aggressive deficit) must all be in place to see FFM during fat loss. Another study in obese older adults used a specialised whey protein, leucine, and vitamin D supplement during a diet/exercise programme and found that while both control and supplemented groups lost ~3 kg of fat, the supplemented group gained ~0.4 kg appendicular FFM whereas the control group lost ~0.5 kg [35]. The supplemental leucine and vitamin D likely helped overcome some anabolic resistance in muscle.

Older adults can accrue skeletal muscle with resistance training, but the mean magnitude and rate of accretion are lower than in young adults. A principal mechanism is anabolic resistance [39] a blunted MPS and translational-signalling response to amino-acid provision and mechanical loading requiring higher per-meal protein/leucine doses and adequately intense resistance exercise to achieve a given MPS response [40]. At the whole-body level, meta-analytic time-course data show modest but significant FFM gains in adults ≥50 y with resistance training (≈1 kg over ~20 weeks on average), with age negatively moderating hypertrophy [41]. Direct comparisons under identical programmes confirm that older adults do increase muscle mass, but to a smaller fibre-level magnitude than young adults [41], consistent with impaired myonuclear accretion and ribosomal biogenesis [42,43]. Their primary endpoint was function rather than pure recomposition, but it highlights that even with optimal interventions, older adults may mainly preserve FFM during fat loss rather than substantially increase it. However, if the training stimulus is novel (many older individuals are untrained) and protein intake is increased from inadequate to high, some lean gains can occur.

In younger adults (20s–40s), anabolic hormones and muscle protein synthetic responses are at their peak. They typically show the greatest absolute gains in muscle and can tolerate higher training volumes, which supports recomposition when diet is aligned [19,41]. Garthe et al. [14] demonstrated that young athletes were able to add muscle in an energy deficit, whereas one would rarely see a 70-year-old gain 1–2 kg muscle while losing fat over a month. Nonetheless, older individuals can improve relative body composition significantly (e.g. lose 5% fat and maintain muscle, effectively increasing percent FFM). Special nutritional adjuncts may help older populations recomposition, such as leucine supplementation, creatine, or HMB, which have shown some efficacy in preserving muscle mass in hypocaloric states [44]. Ensuring sufficiently high protein intake (≥1.2–1.5 g/kg in older adults, sometimes up to ~2.0 g/kg) is recommended because older muscle requires a stronger EAA dose to maximally stimulate MPS [19].

In summary, age modulates recomposition potential primarily through physiological anabolic responsiveness. Young individuals can typically increase skeletal muscle mass and reduce fat mass more readily, whereas older adults require very targeted interventions and often see smaller changes. However, even in older age, positive recomposition (gain strength/muscle, lose fat) is achievable and indeed crucial for healthy aging; it just demands supervised exercise programmes and nutritional support (protein, vitamin D, etc.).

#### 3.4.1. Other modulators (sex and hormones)

On average, men possess greater absolute skeletal muscle mass and higher circulating testosterone concentrations than women; historically, these sex-based differences have been interpreted as implying a greater propensity for body recomposition in men. Men do often have larger absolute gains in muscle and can lose fat more rapidly (due to higher energy expenditure) [2,45]. However, women are capable of recomposition as well. In fact, some studies suggest that women may exhibit superior preservation of fat-free mass relative to total weight loss, potentially due to sexual dimorphism in fuel utilisation, specifically, a greater reliance on lipid oxidation which may spare FFM during energy deficits. For instance, Garthe et al. observed that female athletes gained fat-free mass even during rapid weight loss, whereas males experienced significant FFM losses under identical relative deficit conditions [14]. Taken together, current evidence does not indicate a systematic sex-based advantage for recomposition when training and total protein are matched; meta-analytic work shows protein-augmented hypertrophy responses are broadly similar across adults, with sex not a primary moderator once dosing and training are controlled [45]. Regardless, both sexes respond similarly when equating training and nutrition, with protein needs being universally important.

Hormonal milieu is a key modulator of body recomposition. Higher circulating anabolic hormones (e.g. testosterone, IGF-1) and exogenous androgen use markedly augment hypertrophy and fat-free mass accrual, thereby facilitating recomposition; in controlled trials, supraphysiologic testosterone increased muscle size and strength and showed clear dose–response effects on FFM even without training [24]. In natural athletes, maintaining a favourable endocrine environment includes avoiding excessively low-fat diets which have been associated with reductions in circulating testosterone in intervention studies managing psychological and training stressors that elevate glucocorticoids, and correcting bona fide micronutrient deficiencies [46,47]. In vitamin-D–deficient men, supplementation has increased testosterone in randomised trials [48], and zinc repletion increases testosterone in zinc-deficient adults [49]. Conversely, chronic overreaching and sustained low energy availability can suppress hypothalamic-pituitary-gonadal and thyroid axes and precipitate Relative Energy Deficiency in Sport (REDs) with endocrine, metabolic, and performance sequelae; recent International Olympic Committee (IOC) consensus statements emphasise identification and management in women and men [50,51].

Beyond the primary inputs of training and nutrition, lifestyle variables, specifically sleep sufficiency, act as potent modulators of body composition that are frequently underappreciated in practice. Sleep deprivation creates a catabolic milieu characterised by elevated cortisol and impaired recovery. Notably, a clinical trial by Nedeltcheva et al. [52] demonstrated that participants on a hypocaloric diet who slept only ~5.5 hours per night lost 60% more FFM and 55% less fat than those sleeping 8.5 hours, despite consuming identical diets. [52] This finding suggests that inadequate sleep can negatively influence body recomposition by shifting weight loss towards FFM loss. It should be noted, however, that the study did not include a resistance training component, limiting generalisability of findings. Although evidence remains limited, ensuring sufficient, quality sleep appears to be a mitigating factor for optimising recomposition. Good sleep supports growth hormone release and recovery, conceivably facilitating muscle repair and fat oxidation.

### 3.5. Nutritional interventions for recomposition

Nutrition is the cornerstone of body recomposition, as it provides the substrates for muscle growth and orchestrates the energy balance for fat loss. Several nutritional strategies have strong evidence for facilitating recomposition:

### 3.6. High protein intake

Resistance training is the primary stimulus for FFM accretion and retention during energy restriction; dietary protein chiefly augments the training response [45,53,54]. In physically active individuals, position statements and athlete-focused syntheses support ~1.4–2.2 g·kg^−1^·day^−1^ protein, with higher targets during hypocaloric phases ~2.3–3.1 g·kg^−1^ fat-free mass·day^−1^ to mitigate FFM losses [55,56]. Practically, many recomposition studies have used ~2.0–2.5 g/kg, often translating to 25–35% of total calories from protein [1,4]. These levels are well above the general RDA (0.8 g/kg) but have consistently shown benefits in body composition outcomes without adverse effects in healthy individuals. For example, Antonio et al. [57] had resistance-trained men and women consume a very high protein diet (~3.4 g/kg) for 8 weeks. Despite consuming about 400 calories more per day than a control group, the high-protein group did not increase fat mass (in fact, they slightly lost fat), and they gained a similar amount of FFM as the normal diet group [57]. This suggests that protein intakes at the upper end of the recommended range may confer a protective 'buffer' against fat gain, potentially favoring FFM accretion due to the increased thermic effect of feeding and the metabolic inefficiency of converting excess amino acids to adipose tissue [57]. These mechanistic advantages align with meta-analytic data demonstrating that high-protein energy-restricted diets result in superior fat loss and fat-free mass preservation compared to standard-protein controls [9].

Protein distribution further optimises the adaptive response. Spreading ~0.3-0.4 g·kg^−1^ per meal across 3-5 feedings effectively saturates per-meal MPS pulses, although total daily protein remains the dominant determinant [1,18,53]. In older adults, overcoming anabolic resistance may require larger leucine-rich boluses, approximately ~0.4-0.6 g·kg^−1^ or ~35-40 g high-quality protein, with whey and other leucine-rich proteins producing robust postprandial MPS responses [5,19,58]. Pre-sleep casein can increase overnight aminoacidemia and support nocturnal MPS after evening training [59]. Collectively, recomposition is best achieved by prioritising progressive RT and then aligning total daily protein and per-meal distribution to support training-mediated adaptations [45,54].

### 3.7. Energy intake and deficit size

To lose fat, some degree of energy deficit is required. For recomposition, the deficit often should be moderate, not extreme, to facilitate FFM gains or at least prevent muscle loss. A common strategy is to set a daily deficit of approximately 300–500 kcal (15–25% below maintenance). This tends to yield fat loss of ~0.25–0.5 kg per week in an average adult, which is slow enough to preserve muscle with proper training [14]. Indeed, as discussed, rapid weight loss (>1% of body weight per week) is associated with higher FFM losses, even in well-trained athletes [14]. Energy availability governs the magnitude and direction of body composition change during resistance training. Meta-regression in resistance-trained cohorts indicates that an energy deficit of approximately 500 kcal·day^−1^ is sufficient to abolish gains in FFM during training, supporting the use of smaller, sustained deficits when recomposition is the goal [12,14]. Consistent with this finding, controlled work in athletes shows that slower weight loss (≈0.7% body mass·week^−1^) better preserves FFM and performance than faster loss (≈1.4%·week^−1^) [14]. Practically, individuals with higher adiposity may tolerate a larger initial deficit, but as leanness increases, tapering toward a modest deficit helps maintain training quality and FFM. An alternative is to operate near estimated energy balance while increasing planned activity (e.g. added conditioning) to create a small effective deficit; this can reduce perceived dieting stress but requires monitoring as maintenance needs decline with fat loss. Over the long term, the best partitioning arises from a balance between a modest net deficit and the preservation of training stimulus/quality [14].

### 3.8. Carbohydrate and fat allocation

After establishing total energy and protein targets, the carbohydrate–fat ratio can be individualised to support training quality and adherence. Carbohydrate availability is the principal dietary lever for sustaining high-intensity work and rapid recovery via glycogen resynthesis; low muscle glycogen can reduce the ability to maintain training intensity and increase tissue breakdown during higher-volume sessions (ISSN position stand on nutrient timing; Carbohydrate availability is the principal dietary lever for sustaining high-intensity work and rapid recovery via glycogen resynthesis; low muscle glycogen can reduce the ability to maintain training intensity and increase tissue breakdown during higher-volume sessions. Indeed, primary data demonstrates approximately 39% glycogen depletion following repeated bouts of leg extension exercise, which coincides with decrements in work output [60,61]. Accordingly, consensus guidance recommends moderate carbohydrate intakes for athletes, scaled to training load (e.g. ~3–5 g·kg^−1^·day^−1^ for light–moderate loads, higher with greater volume), with daily totals prioritised over fine timing, and peri-workout carbohydrate used pragmatically to protect session quality and recovery [27].

The paradigm of carbohydrate periodisation, formalised as the 'fuel for the work required' framework, necessitates the dynamic manipulation of carbohydrate availability to align with the specific glycolytic demands of training sessions [15,62]. Theoretically, this strategy optimises glycogen status for high-intensity output while facilitating energy deficits during periods of lower metabolic demand [62]. However, empirical substantiation for the superiority of this approach in body recomposition contexts remains limited; while chronic carbohydrate restriction may compromise resistance training volume and performance [60,63], data confirming that periodised intake yields superior fat-free mass accretion compared to continuous restrictions are equivocal. Consequently, carbohydrate periodisation should be interpreted as a pragmatic strategy to preserve training intensity rather than a definitively proven metabolic necessity for hypertrophy [15,60]. While training with low carbohydrate availability can up-regulate some molecular markers of endurance adaptation, chronic or indiscriminate restriction tends to compromise training quality and high-intensity performance; data in elite athletes also demonstrated impaired exercise economy with ketogenic low-carbohydrate, high-fat strategies despite isoenergetic diets [62] In resistance/physique contexts, reviews indicate that carbohydrate restriction does not uniformly impair all strength outcomes, but lower availability is more likely to limit performance as session volume and density rise [63,64].

When energy is restricted for fat loss, either lower-fat or lower-carbohydrate diets can produce similar 12-month reductions in weight and adiposity when protein is adequate and calories are matched underscoring that adherence and energy balance dominate the macronutrient split per se [27,30]. Nevertheless, very-low-fat diets may reduce circulating testosterone in men (with uncertain effects on performance), so many sport-nutrition statements retain a pragmatic lower bound of ~20% of energy from fat, emphasising unsaturated sources [64].

### 3.9. Nutrient timing and ergogenic aids

Total daily protein intake is the dominant nutritional determinant of hypertrophy and FFM retention; peri-workout timing per se has, at most, a modest additional effect when daily intake is adequate [45,54,55]. To optimise the cumulative MPS response, protein should generally be distributed across 3–5 daily feedings, targeting approximately 0.3–0.4 g·kg^−1^ per meal [18,65]. However, older adults exhibit anabolic resistance and require larger protein boluses to maximally stimulate myofibrillar protein synthesis [19,66]. While pre-sleep casein ingestion (~30–40 g) facilitates overnight MPS, its additive benefit for body recomposition appears negligible when total daily protein intake is sufficiently high [59,67]. Similarly, while immediate post-exercise carbohydrate ingestion accelerates glycogen resynthesis, this strategy is primarily relevant for athletes requiring rapid recovery between multiple daily sessions; over a 24-hour period, total carbohydrate intake relative to training load dictates replenishment [20,60].

Select ergogenic aids may indirectly support nutrient partitioning by enhancing training quality and total volume. Creatine monohydrate possesses strong evidentiary support for enhancing high-power performance and augmenting fat-free mass accrual during training [68,69]. Caffeine is a robust ergogenic aid that enhances performance across multiple modalities; doses of ~3–6 mg·kg^−1^ ingested 30–60 minutes pre-exercise can reduce ratings of perceived exertion and improve motor-unit recruitment, thereby supporting the maintenance of training intensity [70]. Conversely, beta-hydroxy-beta-methylbutyrate (HMB) demonstrates limited efficacy in protein-replete, trained athletes, with potential utility restricted to clinical populations or untrained individuals initiating exercise [5,71]. Finally, Vitamin D sufficiency should be verified, as correcting deficiencies may support muscle function and strength development [72].

### 3.10. Adherence and behavioural factors

From a practical standpoint, the best diet is one the individual can adhere to while meeting protein and energy targets. Recomposition often requires accurate dietary control, but the degree of tracking should match the athlete or client. Apps and weighed records may improve precision, whereas simpler behavioural rules, such as protein at each meal, high-fibre foods, and carbohydrate focused around training, can support adherence when rigorous tracking is impractical. Diet breaks, defined as periods of eating at maintenance for approximately one week or longer, may improve adherence and support short-term performance, although current trials do not consistently show superior body composition outcomes compared with continuous energy restriction [36,73].

In summary, nutritional interventions for recomposition should first establish adequate protein and an appropriate energy target, then distribute carbohydrate and fat to support training quality, recovery, adherence, and health. Supplements may help indirectly when they improve training capacity or correct deficiencies, but they do not replace progressive resistance training, sufficient protein, and a sustainable energy prescription.

### 3.11. Resistance training for hypertrophy and retention

Progressive resistance training (PRT) is essential for eliciting FFM gains and retaining FFM during energy restriction. Without resistance exercise, a meaningful portion of weight loss can come from FFM, especially in individuals dieting aggressively or with low protein intakes [26, 28, 74]. An effective recomposition programme should provide sufficient mechanical tension, volume, and proximity to failure to stimulate hypertrophy while avoiding unrecoverable fatigue during energy restriction [54].

The training volume (sets per muscle per week) might need adjustment during recomposition. Some evidence suggests slightly higher training volumes can maximise hypertrophy when calories are ample [54]. But in a caloric deficit, recovery is compromised, so extremely high volumes could exceed recovery capacity, raise injury risk or lead to muscle breakdown. A reasonable approach is to use a moderate-to-high volume that the individual can recover from given their nutritional support. For example, ~10–20 sets per major muscle group per week might be effective, spread over 2–3 sessions for that muscle [54]. Advanced lifters may manage the higher end; novices respond to the lower end [75]. Regarding volume manipulation during energy restriction, recent randomised controlled data indicates that higher training volumes do not appear to confer additional benefits for FFM retention or muscle thickness compared to moderate volumes [75]. Consequently, increasing volume under the assumption that 'more is better' may yield diminishing returns when energy availability is compromised. Monitoring performance metrics is critical; if strength outcomes consistently decline, training volume or frequency should be systematically reduced to mitigate overreaching.

The magnitude of load, heavier resistance is generally required to recruit high-threshold Type II muscle fibres, which possess superior hypertrophic potential. However, moderate loads (60–75% of 1RM) performed for moderate repetitions (6–12) to voluntary failure also effectively stimulate hypertrophy, suggesting a mixed approach is optimal [55]. Extending this principle, recent evidence demonstrates that the phase-specific application of load, not simply total load magnitude, differentially influences the neuromuscular stimulus: accentuated eccentric loading in the barbell back squat produces distinct concentric and eccentric velocity profiles and kinematic parameters compared to traditional resistance loading, suggesting that manipulating eccentric overload may provide a targeted means of recruiting high-threshold motor units beyond what conventional loading achieves [76]. Crucially, trainees must strive to maintain or incrementally increase training loads over time; the ability to increase resistance or repetition volume during an energy deficit serves as a robust physiological proxy for the preservation or accretion of contractile tissue. Indeed, recent meta-analytic data demonstrates that programmes incorporating periodised progressive overload, systematically increasing neuromuscular demand, yield superior strength and hypertrophic adaptations compared to non-periodised protocols [77]. The subsequent section examines the requisite resistance training stimuli and their integration with these nutritional strategies to optimise body composition outcomes. Figure 3 depicts response surfaces linking protein dose and weekly RT volume to ΔFFM in two contexts. In novices with higher adiposity (left panel), the anabolic “safe zone” is wide, multiple protein–volume combinations yield positive ΔFFM even in an ~18% deficit. In trained/lean athletes (right panel), the ΔFFM = 0 isocline shifts upward/rightward, shrinking the feasible zone and necessitating higher protein and careful volume management under a smaller (~8%) deficit. The gradient vectors emphasise that, in trained/lean states, marginal gains come more from optimising adherence and protein than from simply adding volume.

### 3.12. Concurrent training and cardio

The integration of endurance and resistance training (concurrent training) has historically been postulated to attenuate muscle hypertrophy due to the “interference effect” a phenomenon attributed to competing molecular signalling and residual fatigue [78]. However, recent systematic reviews and meta-analyses indicate that this effect is not uniform across all levels of muscle morphology. While concurrent training does not appear to compromise whole-muscle hypertrophy compared to resistance training alone [79,80], interference may still occur at the myofiber level, particularly in Type I fibres [81]. To optimise body recomposition and maximise hypertrophic potential, the following evidence-based strategies should be employed:

### 3.13. Exercise modality

The biomechanical nature of the aerobic stimulus significantly influences cellular adaptations. Evidence suggests that concurrent training involving running results in a significant attenuation of muscle hypertrophy and power compared to resistance training alone [81]. In contrast, cycling does not induce this negative effect at the fibre level [81]. Therefore, low-impact, concentric-dominant modalities such as cycling may be preferable to running when preserving maximal muscle fibre size.

Intensity and Protocol (HIIT vs. Continuous): Contrary to the premise that high-intensity interval training (HIIT) is inherently muscle-sparing, recent meta-analytic data suggests that HIIT may pose a greater risk to fibre hypertrophy than continuous endurance training. Monserdà-Vilaró et al. [79] observed that resistance training alone induced significantly greater Type I and Type II fibre hypertrophy than concurrent training when HIIT was incorporated (SMD > 0.33), whereas concurrent training using continuous protocols showed no significant difference compared to resistance training alone. Thus, moderate-intensity continuous training may be more appropriate for maximising fibre growth.

### 3.14. Session sequencing

While exercise order (aerobic vs. resistance) does not statistically compromise whole-muscle hypertrophy, performing aerobic and resistance training in the same session significantly attenuates explosive strength gains compared to separating sessions by at least 3 hours [81]. To ensure the maintenance of training intensity and power output, critical components of the hypertrophic stimulus, prioritising resistance training or separating sessions is recommended where feasible.

### 3.15. Training frequency and split routines

An effective approach is to train each major muscle group ~2 times per week. Higher frequencies (3 + times/week per muscle) can work if volume per session is lower, which can help distribute the stimulus and aid recovery. Full-body routines done 3 days/week, or an upper/lower split 4 days/week, or push/pull/legs 5–6 days/week-all can be viable templates for recomposition, as long as weekly volume and intensity are sufficient. Many body recomposition studies with novices use full-body workouts combining resistance and aerobic training in the same programme [3,4,28]. For example, a typical session might involve 8–10 resistance exercises (covering all major muscle groups) followed by 20 minutes of moderate cardio; done 3 times/week along with a high-protein diet, this yields FFM accretion and FM loss in novices [38]. In trained individuals, split routines might allow more focus and volume for each muscle group while managing fatigue. The key is consistency: hitting each muscle regularly and progressively. Figure 2 summarises the attainable trade-offs between fat loss and FFM gain across protein, deficit, and resistance-training (RT) adherence. The Pareto frontier falls in the upper-left quadrant, indicating concurrent fat loss and FFM gains, with the densest cluster at ~2.2–2.8 g·kg^−1^·d^−1^ protein, ~10–20% caloric deficit, and ≥ 80% RT adherence. This mirrors RCTs showing that high protein during moderate energy restriction maximises ΔFFM for a given ΔFM. Practically, prescriptions that deviate toward larger deficits or lower adherence move off the frontier (resulting in fat loss accompanied by compromised FFM retention or accretion) (Figure 3).

### 3.16. Flexibility and NEAT

Non-exercise activity thermogenesis (NEAT), which includes all daily movement outside of formal exercise, can significantly influence fat loss [2,32]. Encouraging an active lifestyle (taking walks, using stairs, staying on one’s feet when possible) helps increase energy expenditure without “exercise stress.” While not a direct training strategy, being mindful of staying active can complement the formal training regimen. It is common for individuals focusing on heavy training to inadvertently reduce NEAT (because they are more fatigued), which can stall fat loss [32]. Therefore, maintaining step counts or including light recreational activities can elevate NEAT, aiding the energy deficit for fat loss without meaningfully interfering with recovery, provided the duration is managed to avoid excessive cumulative fatigue.

### 3.17. Deloads and recovery

To sustain progress, planned light weeks or “deloads” may be beneficial, especially for advanced trainees. During a deload (often 5–7 days), one might substantially reduce training volume and intensity or take a break from weight training entirely. This allows connective tissues to recover and can restore sensitivity to training [82]. In a recomposition context, a deload can coincide with a diet break (eating at maintenance) for psychological and physiological reset. Adequate recovery practices-sleep, nutrition (as covered), stress management-all play into training effectiveness. High stress or insufficient sleep can degrade training quality and recovery, as previously mentioned. Additionally, incorporating specific preparatory strategies (e.g. dynamic warm-ups, foam rolling) can serve to optimise neuromuscular readiness and transiently increase range of motion without compromising force production, thereby supporting movement quality and training consistency [83,84].

### 3.18. Monitoring progress in training

Monitoring training performance serves as a pragmatic, albeit indirect, gauge of physiological adaptation. While strength increases can occur via neuromuscular mechanisms independent of hypertrophy, sustained improvements in load or volume capacity during energy restriction generally signal that contractile tissue is being preserved or accrued. Conversely, if performance consistently stagnates or declines, and this reduction is not attributable to acute fatigue or planned tapering, it potentially indicates overreaching or insufficient energy availability [50]. In a recomposition phase, some minor strength loss might occur if a person is in a steep deficit (especially maximal strength or power might dip due to reduced leverage from fat loss or lower energy availability). But generally, one should aim to maintain or increase strength. Two recomposition studies report improvements in strength even as weight is lost [1,14]. For instance, in Garthe et al. [14], the slow-diet group increased their 1RM squat and bench press significantly while losing weight [14]. Therefore, incorporating progressive strength benchmarks in the programme provides a general gauge that may indicate muscle is being gained over time.

In summary, the exercise component of recomposition should centre on progressive resistance training (PRT), supplemented by aerobic or conditioning work that aids FM loss without compromising FFM accretion or retention. A practical programme may include 3-5 days of resistance training and 2-3 days of low-impact cardio or conditioning, adjusted for training status, recovery, sleep, and rate of weight loss. Figure 4 visualises inter-individual variability under a standardised periodised plan and supports iterative adjustment rather than a fixed prescription.

### 3.19. Periodisation models and practical algorithms for recomposition

While some individuals can intuitively progress by consistently training and eating well, a formal periodisation—the systematic planning of training and nutrition phases—may help to optimise recomposition over the long term. Periodisation traditionally refers to altering training variables over defined periods to peak for performance; for recomposition, we extend the concept to coordinating both training and diet in phases that maximise FFM, maximise fat loss, or aim for both, in a logical sequence.

### 3.20. Traditional vs. concurrent periodisation

A traditional approach to body composition is sequential: bulk then cut. For example, an athlete might spend 4–6 months in a caloric surplus focusing on muscle and strength gains (accepting some fat gain), then follow with a 2–3 month cutting phase to reduce fat (accepting minimal FFM or some muscle loss). Over a multi-year cycle, this can yield large muscle increases but at the cost of cyclical weight changes.

In contrast, a concurrent recomposition approach attempts to increase FFM or skeletal muscle mass and reduce FM continuously or in short cycles. This can involve mild, alternating phases of small surplus and small deficit, or weekly non-linear energy intake in which higher energy and carbohydrate availability are placed on demanding training days and lower energy intake is used on recovery days. Such strategies should be viewed as adherence and performance tools rather than guaranteed metabolic advantages.

### 3.21. Nutritional periodisation

Nutritional periodisation in recomposition often involves refeeds or diet breaks. A refeed is a 1–3-day period of increased energy intake, typically from carbohydrate, during an extended deficit. Diet breaks are longer maintenance-energy periods. Current evidence indicates that these strategies may support adherence, hunger management, and short-term training quality, but they do not consistently produce superior FM loss, FFM retention, or resting metabolic rate outcomes compared with well-matched continuous energy restriction [36,73,85].

For advanced athletes, contest preparation often employs highly refined periodised approaches, typically initiating with a moderate deficit and subsequently incorporating non-linear strategies such as refeeds or diet breaks. While such protocols are frequently implemented in practice to mitigate hormonal suppression and psychological fatigue [6,34], it is important to note that support for these practices is primarily derived from observational studies and case reports. Current experimental evidence confirming their physiological superiority over continuous restriction for preserving FFM in contest-lean cohorts remains limited. The final weeks might include peaking strategies (like water/carbohydrate manipulation) which are beyond our scope, but the core is that the fat loss is done gradually enough to keep muscle, often over 3–6 months.

### 3.22. Training periodisation

On the training side, periodisation can be used to emphasise different aspects at different times, which can indirectly assist in achieving recomposition. For example, a macrocycle might have a hypertrophy-focused phase (higher volume, moderate intensity) followed by a strength-focused phase (higher intensity, moderate volume). The hypertrophy phase could align with slightly higher calories to maximise FFM, and the strength phase could align with a small deficit to tap into fat stores while enhancing neuromuscular gains. Because strength training is neutrally demanding but often lower volume, some people find they can reduce calories during a strength phase without losing muscle because the stimulus to maintain muscle (lifting heavy) is still present and fatigue is lower, allowing recovery on fewer calories. On the other hand, the high-volume hypertrophy work might require more calories to recover and build tissue, so pairing with at least maintenance or slight surplus calories is logical. Over the course of the periodised plan, the net effect can be recomposition [86].

Another periodisation model involves an undulating approach in which sessions target strength, hypertrophy, and conditioning qualities across a week or mesocycle. For example, an athlete might perform heavy low-repetition work early in the week, moderate-repetition hypertrophy work mid-week, and a metabolic circuit or HIIT session later in the week if recovery permits. This approach can develop multiple fitness qualities, but the independent hypertrophic benefit of periodisation is uncertain when volume is equated. Meta-analytic evidence indicates clearer advantages for maximal strength than for hypertrophy per se, reinforcing the need to match periodisation to the primary outcome and recovery state [77,87,88].

**Figure 2.**
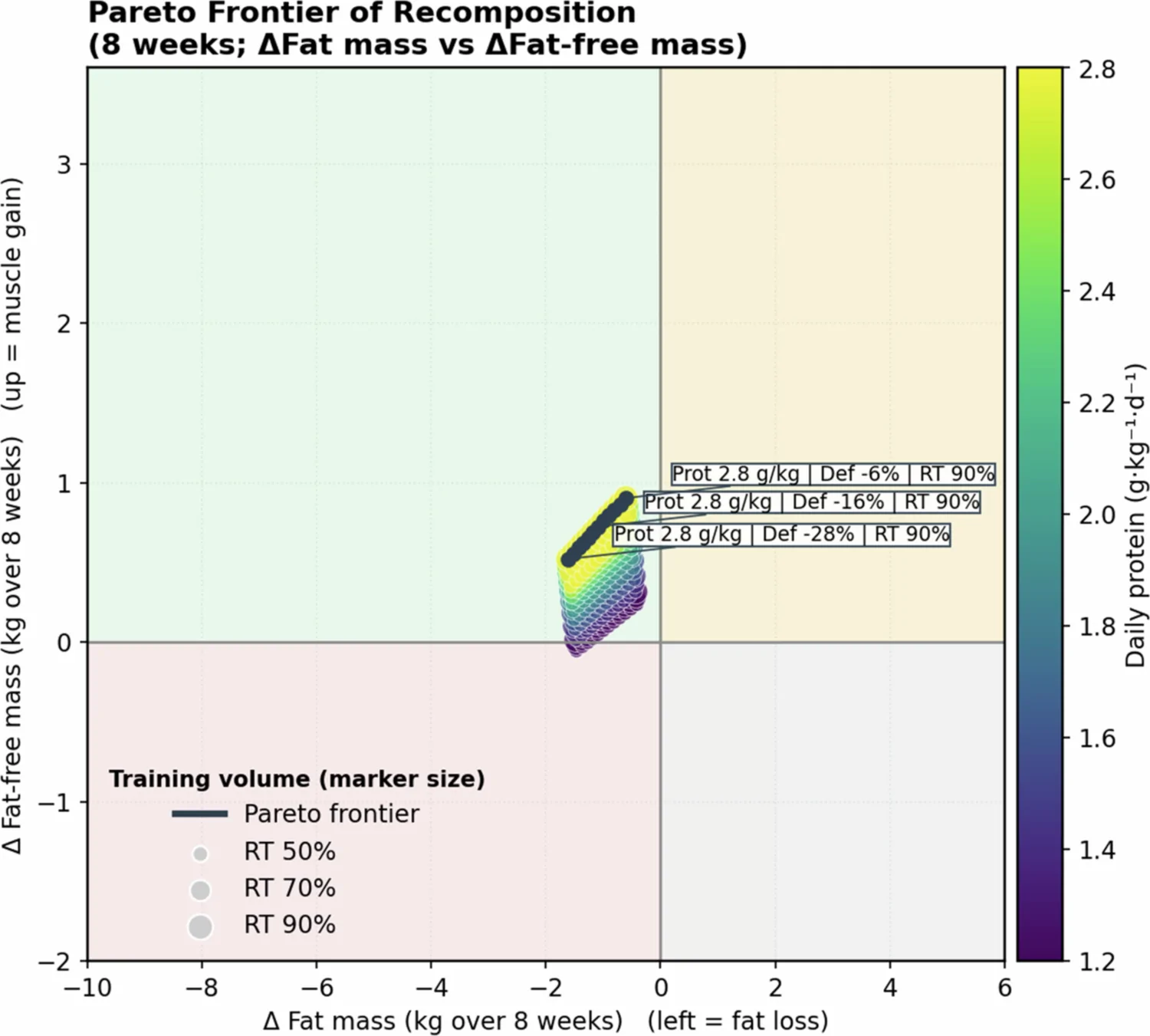
Pareto frontier of body recomposition over 8 weeks (ΔFat mass vs ΔFat-free mass). Each marker represents a feasible prescription generated from combinations of daily protein (1.2–2.8 g·kg^−1^·d^−1^; colour scale), energy deficit (≈6–28%), and resistance-training (RT) adherence/volume (marker size; 50–90% of target weekly sets). The thick polyline connects non-dominated solutions (Pareto frontier) that maximise ΔFFM for any given fat loss. Leftward movement indicates greater fat loss (ΔFM < 0); upward movement indicates FFM (ΔFFM > 0). Shaded quadrants highlight goal regions (upper left = concurrent fat loss and FFM). Callouts illustrate representative high protein/deficit settings at high RT adherence. In this simulation, the best trade-offs cluster around protein ≈2.2–2.8 g·kg^−1^·d^−1^, moderate deficits ≈10–20%, and RT adherence ≥80%, consistent with RCTs showing FFM preservation/gain with high protein under energy restriction. Abbreviations: ΔFM, change in fat mass; ΔFFM, change in fat-free mass; RT, resistance training.

**Figure 3.**
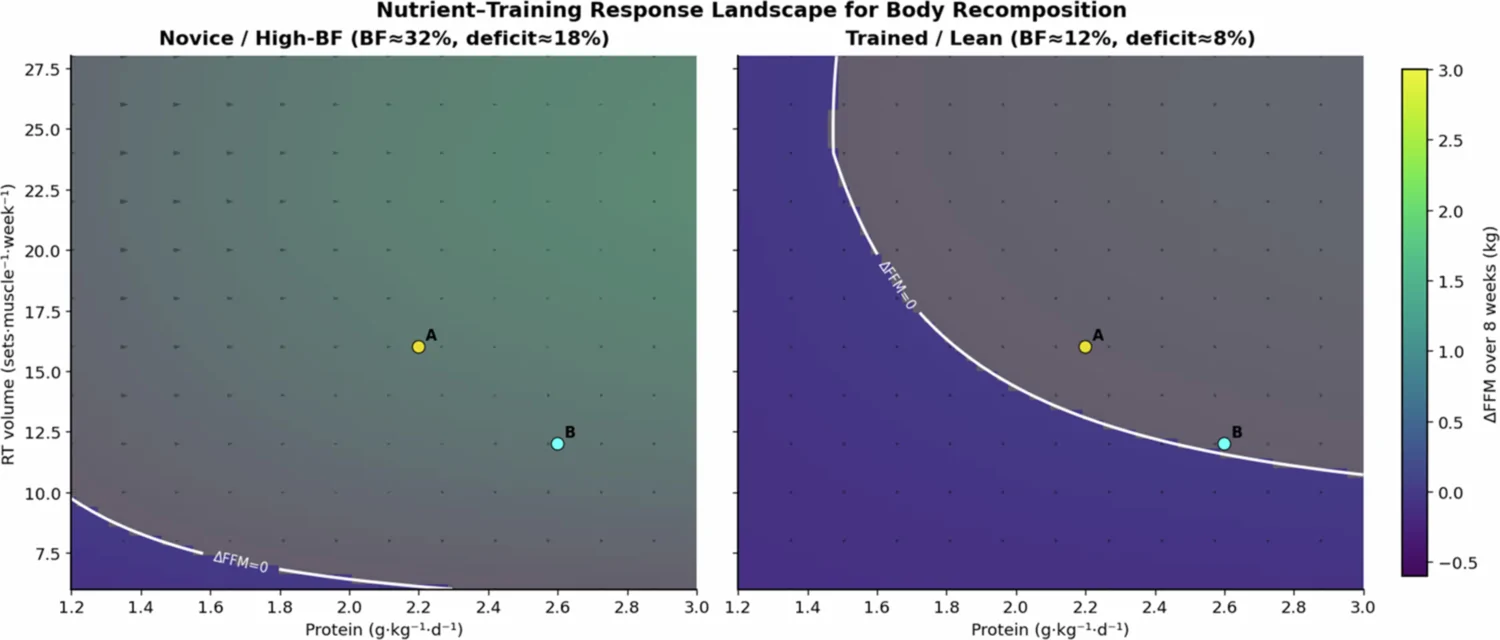
Nutrient-training response landscapes for recomposition in (left) novice/high-adiposity and (right) trained/lean athletes. Heatmaps show modelled ΔFFM (kg over 8 weeks; colour bar) across daily protein intake (x-axis, 1.2–3.0 g·kg^−1^·d^−1^) and RT volume (y-axis, weekly sets per muscle ≈8–28). White contour denotes the ΔFFM = 0 isocline (boundary between expected loss vs gain of FFM). Small arrows depict the local gradient (direction of steepest expected improvement in ΔFFM). Scenario assumptions: novice/high-BF panel uses an ≈18% deficit; trained/lean panel uses an ≈8% deficit; carbohydrates are preferentially allocated to heavy RT days; sleep and NEAT are held at typical targets. Points A and B illustrate plausible prescriptions (e.g., A ≈2.2 g·kg^−1^·d^−1^ with moderate-high RT volume; B ≈2.6 g·kg^−1^·d^−1^ with lower volume). The anabolic “safe zone” (above the isocline) is broad in novices and narrow in trained/lean athletes, emphasising the need for higher protein, careful volume management, and smaller deficits as training status advances. Abbreviations: RT, resistance training; NEAT, non-exercise activity thermogenesis.

**Figure 4.**
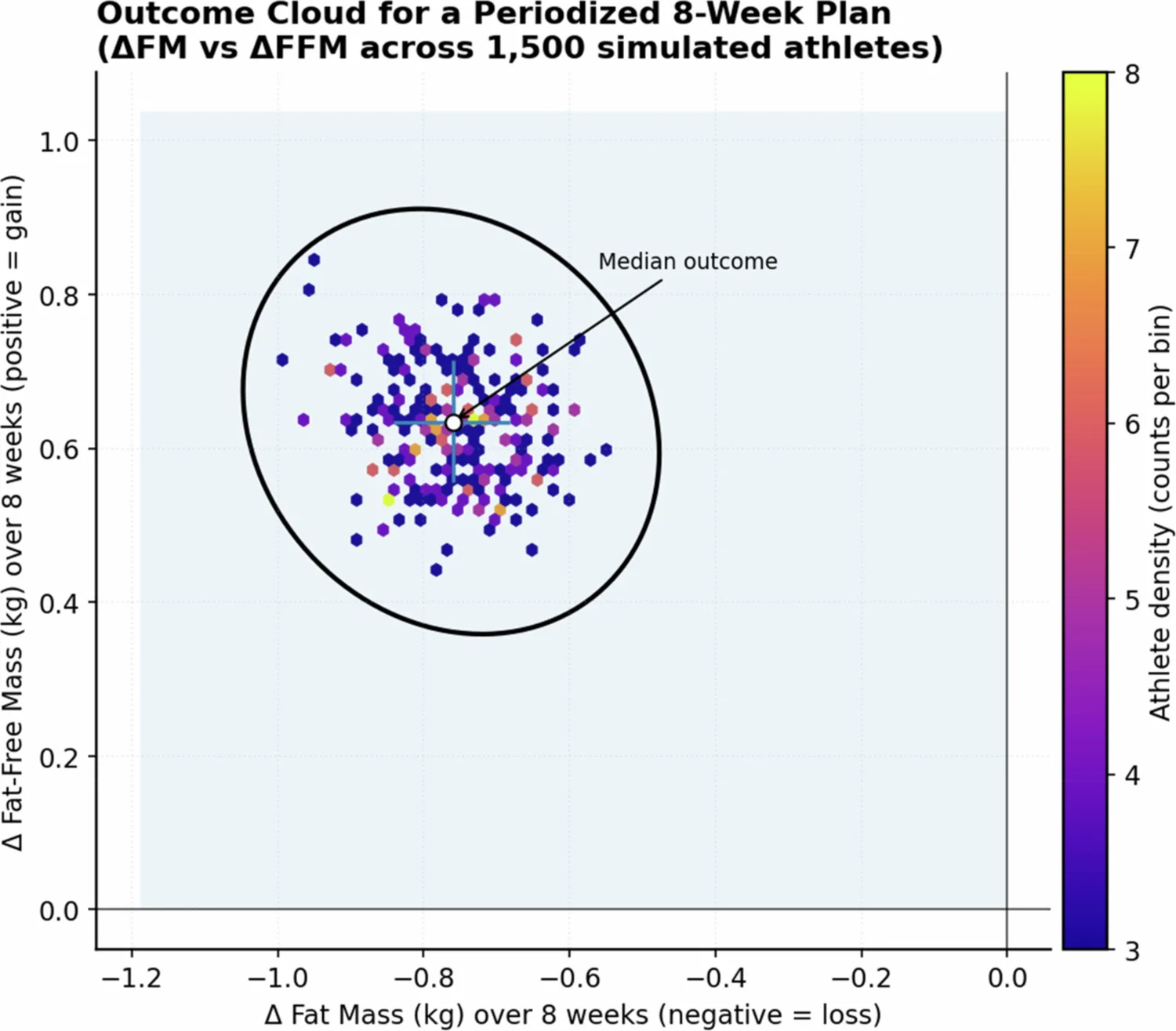
Outcome cloud for a periodised 8-week plan across 1,500 simulated athletes (ΔFM vs ΔFFM). Each point is one athlete’s outcome under a standardised plan (progressive RT 3–5 d·wk^−1^, HIIT 1–2×·wk^−1^ as tolerated, protein target ≈2.2 g·kg^−1^·d^−1^, moderate deficit, carbohydrate periodisation). Colour encodes local observation density; the black ellipse depicts the ~68% probability contour of the bivariate distribution. Crosshairs mark zero-change axes. The median outcome (white diamond) lies in the upper-left quadrant (typical pattern: modest ΔFFM gain with concurrent ΔFM loss), while the spread reflects adherence, sleep, baseline adiposity, and training status variability. This figure communicates *heterogeneity of response* and supports individualised adjustment of deficit, protein, and RT volume. Abbreviations: ΔFM, change in fat mass; ΔFFM, change in fat-free mass; RT, resistance training; HIIT, high-intensity interval training.

For an intermediate resistance-trained adult with ~15% body fat seeking gradual body recomposition, we propose a 12-week periodised plan coordinating energy intake and training emphases. Phase 1 (Weeks 1–4; hypertrophy focus, mild deficit)**:** Energy intake is set at ~10% below estimated maintenance (targeting ~0.3 kg·wk^−1^ fat loss). Daily protein is ~2.2 g·kg^−1^ with an even distribution across 3–5 meals. Resistance training emphasises higher volume (approximately 12–15 working sets per muscle group per week) with a primary repetition range of 8–15 to drive hypertrophic adaptations. Two brief HIIT sessions (e.g. 10–15 min each) are included weekly to augment energy expenditure while preserving training quality. Strength should be monitored; stable or modestly improving loads/reps indicate adequate recovery and nutrient partitioning. Note that the proposed model is presented as a general template that should be customised to individual needs and responses.

**Week 5 (diet break + deload):** Energy returns to maintenance, with a relative carbohydrate increase to support glycogen repletion. Training volume is reduced by ~50% (intensity largely retained) to facilitate connective-tissue recovery and restore readiness.

**Phase 2 (Weeks 6–9; strength focus, continued recomposition):** Energy returns to a modest deficit (~15% below maintenance, scaled to response after Phase 1). Training shifts toward heavier loading (primary sets in the 4–6 rep range for compound lifts) with slightly lower total volume, while key hypertrophy accessories are maintained. If heavy loading is compromised by HIIT, substitute 1–2 short steady-state sessions instead (e.g. 20–30 min) to sustain the caloric gap. The desired rate of change is ~0.25 kg·wk^−1^ fat loss with maintenance or improvement of 1RM-adjacent performance.

**Week 10 (conditional diet break/deload):** If objective or subjective fatigue is evident (stalled performance, poor sleep, rising soreness), implement a second maintenance-calorie week and a reduced-volume microcycle; otherwise, proceed.

**Phase 3 (Weeks 10–12; hypertrophy tune-up or metabolic emphasis):** Based on responsiveness and fatigue, either (a) re-increase volume modestly to consolidate hypertrophy at a slight deficit or maintenance, or (b) employ one to two metabolic circuits/HIIT sessions per week to remove residual fat while preserving key strength exposures. Across all phases, adjustments are data-driven: if fat loss stalls for ≥ 2 weeks, increase weekly energy deficit slightly (e.g. −100 to −200 kcal·d^−1^) or add ~10–20 min low-impact cardio; if strength regresses or fullness/“flatness” emerges, prioritise peri-workout carbohydrate, reduce HIIT frequency, or raise calories by ~150–250 kcal·d^−1^.

### 3.23. Monitoring and adjustments

A crucial part of any periodised plan is monitoring progress and adjusting accordingly. For recomposition, tracking body weight, body composition estimates, circumferences, and strength metrics provides valuable feedback for prescription. Because body mass may remain stable while FFM increases and FM decreases, relying solely on scale weight is insufficient. Regular body composition assessments, such as skinfold measurements or DXA scans when feasible, can help quantify compartment-specific change and guide adjustments [89]. Indirect body composition methods, including DXA, BIA, and skinfolds, may lack precision for subtle individual-level changes; therefore, results should be interpreted alongside performance and clinical indicators [16,17].

In summary, periodisation for recomposition involves planned cycling of diet and training emphases to sustain progress while protecting training quality and adherence. Whether using short refeeds, longer diet breaks, or alternating mesocycles, changes should be guided by objective trends in ΔFM, ΔFFM, strength, recovery, and health markers rather than by a fixed template.

### 3.24. Future research directions

Despite growing interest in body recomposition, controlled empirical evidence directly testing integrated FM-loss and FFM-accretion protocols remains limited. Practitioners therefore must still extrapolate from weight-loss, hypertrophy, and athlete nutrition studies. To move from theoretical frameworks to more definitive clinical and performance guidelines, future trials should test the combined manipulation of energy intake, protein dose, resistance training, aerobic training, sleep, and monitoring strategy within the same study designs.

Long-term sustainability: Most recomposition studies span a few weeks to months. Long-term effects (over years) are less documented. Future longitudinal studies could assess whether continuously aiming for recomposition (instead of bulk/cut cycles) equal or better net FFM over time and how it impacts metabolic health markers.

**Optimal protein dosing in specific contexts:** While high protein is endorsed, the upper limit and timing specifics for different populations (e.g. older women vs. young men, or athletes in heavy training) could be refined. Is there additional benefit to going from 2.0 to 3.0 g/kg in experienced athletes during a deficit? For example: *Preliminary work in athletes undergoing energy restriction with resistance training suggests that intakes within current recommendations (~1.2–1.7 g·kg^−1^) may be sufficient to preserve FFM and performance, which underlines the need for dose-response trials [90].* Large-scale RCTs varying protein doses during caloric restriction in athletes could clarify dose-response effects on body composition.

Nutrient timing and cycling strategies: More controlled trials on approaches like carbohydrate cycling, intermittent fasting vs. traditional continuous dieting for recomposition, or the inclusion of refeed days would help substantiate those practices. For example, does a weekly 24-hour refeed significantly preserve FFM compared to a continuous moderate deficit? The data on this is still emerging.

Concurrent training modalities: Further research on how to optimally combine resistance and endurance training for recomposition without interference is valuable. Variables such as different HIIT protocols, timing of cardio relative to lifting (morning vs. evening), and the minimal effective dose of cardio for FM loss in the presence of resistance training could be examined in various populations. Training status may also modulate interference: a meta-analysis found that concurrent training impaired lower-body 1RM strength in trained, but not moderately trained or untrained, individuals, particularly when endurance and resistance work were performed in the same session [91].

Psychological and behavioural factors: Adhering to recomposition protocols can be mentally demanding because changes are slower and require consistent effort. Behavioural science input, e.g. determining which monitoring feedback (weekly DXA scans vs. daily weigh-ins vs. periodic photo comparisons) best keeps individuals motivated and on track, could improve programme design. Also, exploring whether recomposition approaches reduces the risk of disordered eating compared to extreme bulking and cutting cycles might be worthwhile, given the emphasis on balance and sustainability.

**Population-specific interventions:** Tailored research in special populations like postmenopausal women, individuals with metabolic syndrome or diabetes, or adolescent athletes undergoing growth could produce specialised recomposition recommendations (considering unique hormonal milieu, growth considerations, comorbidities, etc.). Different demographic groups may require different training/nutrition tweaks for optimal recomposition results. Example may include older adults, for whom resistance-based circuit training has been proposed as a time-efficient way to improve strength, cardiorespiratory fitness and body composition [92], and female physique athletes, in whom a prospective case study reported fat-free mass gains alongside fat loss during preparation, but impaired neuromuscular performance that persisted after the competitive season [93].

Physiological mechanisms: Future research should explore the molecular and cellular underpinnings of recomposition. For instance, studies examining gene expression changes, muscle fibre type adaptations, satellite cell activation, and other muscle remodelling signals under simultaneous FFM accretion and FM loss conditions could shed light on how the body orchestrates these dual changes. Understanding these mechanisms may reveal targets to enhance recomposition (e.g. nutrient signalling pathways, myokines, adipokines).

Individual variability (responders vs. non-responders): Investigating why some individuals show robust recomposition while others do not (even with similar programmes) is important. Studies that examine genetic polymorphisms, hormone responses, or metabolic profiles in “responders” vs. “non-responders” to recomposition programmes could elucidate factors that enable or hinder simultaneous FFM accretion and FM loss. This could lead to more personalised approaches, identifying who is a good candidate for recomposition and who might need alternative strategies.

## 4. Conclusion

Body recomposition, operationalized as ΔFFM > 0 with ΔFM < 0 during the same intervention period, is challenging but achievable when progressive resistance training, sufficient protein, appropriate energy availability, and recovery are integrated. The strongest and most consistent responses occur in untrained individuals and adults with higher adiposity; trained, lean, and older adults can also improve body composition, but expected changes are smaller and require tighter control of energy deficit, protein intake, training quality, and fatigue.

Practical programming should begin with a moderate energy deficit or near-maintenance intake, protein intake typically in the range of ~1.6-2.4 g·kg^−1^·d^−1^ and higher relative intakes in lean or energy-restricted athletes, and progressive resistance training that preserves or improves strength. Carbohydrate allocation, aerobic work, refeeds, and diet breaks should be used primarily to support training quality, adherence, and recovery rather than as stand-alone recomposition mechanisms.

Interpretation of recomposition outcomes requires appropriate measurement. DXA and multi-compartment models provide stronger whole-body inference than less standardised field methods, but even DXA should be interpreted with attention to least-significant-change thresholds, hydration, recent exercise, and device-specific error. Practitioners should therefore combine body composition measures with strength, performance, waist circumference, sleep, appetite, and other indicators of recovery. Overall, recomposition is best viewed as an individualised, monitored process rather than a universal promise of simultaneous large gains in skeletal muscle mass and large losses of FM.
